# Multiple Insecticide Resistance: An Impediment to Insecticide-Based Malaria Vector Control Program

**DOI:** 10.1371/journal.pone.0016066

**Published:** 2011-01-12

**Authors:** Delenasaw Yewhalaw, Fantahun Wassie, Walter Steurbaut, Pieter Spanoghe, Wim Van Bortel, Leen Denis, Dejene A. Tessema, Yehenew Getachew, Marc Coosemans, Luc Duchateau, Niko Speybroeck

**Affiliations:** 1 Department of Biology, Jimma University, Jimma, Ethiopia; 2 Department of Environmental Health, Jimma University, Jimma, Ethiopia; 3 Department of Chemistry, Jimma University, Jimma, Ethiopia; 4 Department of Plant Science, Jimma University, Jimma, Ethiopia; 5 Department of Crop Protection Chemistry, Ghent University, Ghent, Belgium; 6 Department of Parasitology, Institute of Tropical Medicine, Antwerp, Belgium; 7 Department of Physiology and Biometrics, Ghent University, Ghent, Belgium; 8 Institute for Health and Society, Public Health School, Université Catholique de Louvain, Brussels, Belgium; New Mexico State University, United States of America

## Abstract

**Background:**

Indoor Residual Spraying (IRS), insecticide-treated nets (ITNs) and long-lasting insecticidal nets (LLINs) are key components in malaria prevention and control strategy. However, the development of resistance by mosquitoes to insecticides recommended for IRS and/or ITNs/LLINs would affect insecticide-based malaria vector control. We assessed the susceptibility levels of *Anopheles arabiensis* to insecticides used in malaria control, characterized basic mechanisms underlying resistance, and evaluated the role of public health use of insecticides in resistance selection.

**Methodology/Principal findings:**

Susceptibility status of *An. arabiensis* was assessed using WHO bioassay tests to DDT, permethrin, deltamethrin, malathion and propoxur in Ethiopia from August to September 2009. Mosquito specimens were screened for knockdown resistance (*kdr*) and insensitive acetylcholinesterase (ace-1^R^) mutations using AS-PCR and PCR-RFLP, respectively. DDT residues level in soil from human dwellings and the surrounding environment were determined by Gas Chromatography with Electron Capture Detector. *An. arabiensis* was resistant to DDT, permethrin, deltamethrin and malathion, but susceptible to propoxur. The West African *kdr* allele was found in 280 specimens out of 284 with a frequency ranged from 95% to 100%. Ace-1^R^ mutation was not detected in all specimens scored for the allele. Moreover, DDT residues were found in soil samples from human dwellings but not in the surrounding environment.

**Conclusion:**

The observed multiple-resistance coupled with the occurrence of high *kdr* frequency in populations of *An. arabiensis* could profoundly affect the malaria vector control programme in Ethiopia. This needs an urgent call for implementing rational resistance management strategies and integrated vector control intervention.

## Introduction

Indoor Residual Spraying (IRS), insecticide-treated nets (ITNs) and long-lasting insecticidal nets (LLINs) are pillars in malaria prevention and control strategy in Ethiopia and are being used on a large scale. IRS has been in use for more than four decades [1] and DDT (organochlorine) remains the insecticide of choice for IRS followed by malathion (organophosphate) with a limited application as an alternative insecticide in the country. DDT was being formulated and applied in Ethiopia for public health use only [2]. Each year, more than one million houses were sprayed in about 5,000 localities of the country protecting about five million people from the risk of malaria [3]. On average, from 2000 to 2005, 273,787 kg/year of DDT (75% WDP) was sprayed [4]. An average of 31,638 kg/year of malathion was also used in IRS from 2003 to 2005 by the national malaria control programme (NMCP) of Ethiopia (G. Tesfaye personal communication). The IRS coverage also increased from 17% in 2005 [5] to 20% in 2007, targeting 4.2 million households [6]. DDT use for IRS in Ethiopia had been discontinued in favor of deltamethrin in 2009. The use of ITNs was also adopted by Ethiopia in 1997/1998 with the support of World Health Organization (WHO) in selected malarious areas [7]. In 2000, the overall coverage of ITNs was 1–2% [8] and reached 6.4% in 2005 [5]. The distribution of ITNs/LLINs scaled up to almost 20 million between 2006 and 2008 targeting 40 million people at risk [2]. WHO and other public health organizations emphasized the use of pyrethroid-impregnated bed nets for malaria control [9], [10] and WHO has also been promoting the use or reintroduction of DDT for IRS [11]–[13].

However, resistance of mosquito vectors to insecticides is one of the major challenges facing malaria vector control programme [14]–[15]. Particularly, the development of resistance to pyrethroid and DDT by *Anopheles gambiae s.s* and *An. arabiensis*, the two important malaria vectors within the *gambiae* complex in Africa, is a threat to vector control programme. Studies done in sub-Saharan Africa showed wide spread resistance of DDT and pyrethroid in the populations of *An. gambiae s.s* [16]–[18] and *An. arabiensis* [19]–[20]. Likewise, organophosphate and carbamate resistance were documented in *An. arabiensis* [21]–[23] and *An. gambiae s.s* [24]–[27].

In Ethiopia, *An. arabiensis*, the most important malaria vector in the country [28], is strongly resistant to DDT and pyrethroids [29]–[31]. Furthermore, West African knockdown resistance (*kdr*) with allelic frequency of 98.5% was reported in population of *An. arabiensis* from southwestern Ethiopia [31]. Nevertheless, the susceptibility levels of population of *An. arabiensis* to alternative insecticides, the association of the reported high *kdr* frequency with the resistance phenotype, and the occurrence of other possible mechanisms of resistance are poorly understood. Besides, there is no documented data on the relative impact of public health use of insecticides (especially DDT) in resistance development.

In this study, we investigated susceptibility status of malaria vectors to DDT, Pyrethroids, and alternative insecticides (Organophosphates and Carbamates), possible resistance mechanisms (*kdr* and modified acetylcholinesterase mutations) involved in insecticide resistance, the association of *kd*r mutation with resistance phenotype, and the role of public health use of insecticides on the development of resistance in populations of *An. arabiensis*.

## Materials and Methods

### Study area

The study was conducted in the framework of a longitudinal study on malaria incidence and transmission in two groups of villages found in four districts (Omo Nada, Kerssa, Tiro Afeta and Sekoru) surrounding the Gilgel-Gibe hydroelectric dam, southwestern Ethiopia. Villages which are within 3 km distance from the dam were considered as ‘high-risk’ for malaria whereas villages 5–8 km away from the dam were assigned as ‘low-risk’ villages. Detailed description of the selection process of the study villages was described elsewhere [32]. The study area lies between latitudes 07°42′50″ N and 07°53′50″N and between longitudes 37°11′22″E and 37°20′36″E, at an altitude of 1,671–1,864 meter above sea level. It has a sub-humid, warm to hot climate with a mean annual temperature of 19°C and mean annual rainfall is between 1,300 and 1,800 mm. Malaria transmission in the area is unstable and seasonal. Malaria vector control intervention, in the study area, is similar to other parts of the country relying on IRS with DDT/malathion and use of ITNs/LLINs. The primary economic activity of communities in both groups of villages is subsistence farming.

### Mosquito collection, identification and bioassays

Anopheline mosquito larvae were collected by dipping from a range of breeding sites (road puddies, brick pits, pools, marshes, streams, surface water harvest, ditches, dam reservoir shore, pits dug for plastering traditional tukuls, and pits dug for pot making) in the two groups of villages (‘low risk’ and ‘high risk’) during the wet season from July to September 2009. The larvae were reared to adults in the field laboratory at Asendabo Health Centre, southwestern Ethiopia, under standard conditions (25±2°C, 80%±4% (Relative Humidity). Two to three days old, non blood-fed female mosquitoes were selected and exposed to insecticide impregnated papers with discriminating concentrations of DDT (4%), permethrin (0.75%), deltamethrin (0.05%), malathion (5%) and propoxur (0.1%) using WHO standard assays [33]. The insecticide impregnated and control papers were obtained from the WHO collaboration Centre, Vector Control Research Unit, School of Biological Sciences, Penang, Malaysia. Batches of 20–24 mosquitoes in five replicates were exposed in test kit tubes for all bioassays for one hour against the four classes of insecticides and knockdown was recorded at 10, 15, 20, 30, 40, 50, and 60 minutes. Equal numbers of mosquitoes were exposed to the corresponding control papers impregnated with resila oil (Organochlorine control), olive oil (Organophosphate/Carbamate control), and silicone oil (Pyrethroid control). After one hour, mosquitoes were transferred into holding tubes and provided 10% sucrose solution with cotton pads. Mortality was recorded after 24 hours of exposure. Likewise, a strain of *An. arabiensis* from Malaria Training Centre, Nazareth, Ethiopia that has been maintained in the laboratory without exposure to insecticides for over 30 years was exposed to the insecticide papers as reference. The identification of mosquitoes was conducted morphologically as *An. gambiae s.l* using a standard key [34]. Mosquitoes both dead and alive were individually preserved in Eppendorf tubes over silica-gel for further molecular assays.

### DNA extraction, molecular identification, *kdr* and ace-1^R^ mutations detection

Sub-samples of mosquitoes killed and surviving the bioassays were randomly selected per village group; locality and insecticide tested using STATA 11 software (STATA Corp, College Station, TX). Genomic DNA extraction from these sub-samples of alive and dead mosquitoes was carried out by using the procedure described in Collins et al. [35]. DNA was re-suspended in 25 ml sterile TE-buffer (10 mM Tris–HCl pH 8, 1 mM EDTA). Molecular identification of *An. gambiae s.l* was carried out using polymerase chain reaction (PCR) techniques including the primers for *An. gambiae s.s.*, *An. arabiensis* and *An. quadriannulatus A* and *B* following the method used by Hunt et al. [36] and adapted as described in Yewhalaw et al. [31]. The protocol used for the detection of the West African *kdr* (L1014F) and East African *kdr* (L1014S) alleles by allele-specific polymerase chain reaction assay (AS-PCR) was adapted from established protocols [37]–[39]. Assays were also conducted on mosquitoes exposed to malathion and propoxur to detect the presence of insensitive acetylcholinesterase mutations (ace-1^R^ or G119S) using polymerase chain reaction restriction fragment length polymorphism diagnostics (PCR-RFLP) following the methods described in Weill et al. [40] and modified by Djogbenou et al. [41]. Samples of specimens from the laboratory strain of *An. arabiensis* from the Malaria Training Centre, Nazareth, Ethiopia were assayed as well to detect the presence of *kdr* and ace-1^R^ mutations.

### Soil sample collection and preparation

From August to September 2009, indoor and environmental soil samples were collected from seven study villages (4 ‘high risk ’villages, 2 ‘low risk’ villages, and one control village). Indoor residual spraying of DDT has been done in both ‘high risk’ and ‘low risk’ villages but with irregularities in ‘low risk’ villages. The indoor soil samples were taken from wall and floor surfaces of houses in which monthly mosquito collections were conducted from 2007 to 2009 for the longitudinal malaria incidence and transmission study. In each house, 6 samples (3 from the wall, and 3 from the floor surfaces) were taken according to the following procedure. A wooden plate of 20 cm×20 cm, and 30 cm×30 cm was prepared to measure the surface area of the wall and the floor, respectively. The three sampling points, on the wall, were at equal distance from each other and at the middle of the height of the wall. These sampling points were measured and indicated with a marker. Similarly, sampling points on the floor were measured and indicated. The two sampling points were nearly at the center of the floor of the living room and bed room, and the third was adjacent to the wall of the living room. Surface soil layer from these sampling points (3×400 cm^2^ of the wall, and 3×900 cm^2^ of the floor) were scrapped off at a depth of 0.5 cm using a blade adjusted to 0.5 cm. The amount of soil taken from the floor was greater than the wall because it was supposed that there would be less DDT on the floor than on the wall sample as residents clean the floor surface at least once per day and frequently plaster the floor with cow's dung. Moreover, three environmental soil samples were taken at 15 meter distance from the house in three directions with approximate equal angles between each other at a depth of 1–2 cm using a shovel. In between each sampling, the blade and the shovel were washed with water and dried with soft paper before next use. Soil samples collected from a village in Tiro Afeta district with no history of IRS were used as control samples.

The collected soil samples were prepared for further sub-sampling. Samples from the three sampling points of the wall were mixed and made as one composite sample in a plastic bag, and the same was done for the floor and environmental soil samples. Each of the composite samples was coded and weighed. The samples were air dried, grinded and sieved with 1 mm mesh size sieve. Then, 50 gram sub-sample was taken from each of these homogenized soil samples and kept in to a refrigerator until analysis.

### DDT extraction and analysis

DDT extraction and analysis was done at the Department of Crop Protection Chemistry, Faculty of Bioscience Engineering, Ghent University, Belgium. Liquid phase extraction of DDT was done using n-hexane and analysed by Gas Chromatography with electron capture detector (Agilent Technologies 6890 N). Samples of 1 µl were injected using an auto sampler equipped with 10 µl size syringe in to capillary inlet with a glass liner in the split mode. The column was a HP-5 MS 5% phenyl Methyl Siloxane coated capillary column (30 meter length and 250 µm internal diameter). The inlet temperature was set at 280°C and the detector at 320°C. Helium and Nitrogen were used as a carrier and make up gas, respectively. This method was validated for all parameters such as the linearity of the standard series, recovery, and repeatability. The limit of detection and limit of quantification of the instrument was 0.000036 µg/ml and 0.00012 µg/ml, respectively. The standard DDT with 97.2% purity was obtained from Supelco in USA delivered by Sigma-Aldrich logistic Gmbh, Germany.

### Statistical analysis

Data were analysed using descriptive and inferential statistics. Fifty and ninety five percent knockdown times (KDT_50_ and KDT_95_) were computed using logistic regression models using STATA 11 software (STATA Corp, College Station, TX). Fisher's Exact Test was employed to determine the difference in mosquito mortality rates between the two groups of villages for each insecticide treatment, test the association between *kdr* genotype and resistance phenotype, and to test deviation from Hardy-Weinberg equilibrium and population differentiation. As insects in the same tube share common “tube-related” characteristics, we included “tube” as a clustering effect in the calculation of the confidence intervals for the mortalities. The clustering effect was taken into account by applying the Taylor series linearization variance estimation for complex survey data using svy: commands in the STATA 11 software (STATA Corp, College Station, TX). Moreover, t-test was used to determine the significance of the difference between the mean concentration of DDT on the walls, and floors of houses sprayed in June 2008 and 2009 consecutively, and those sprayed only in June 2008.

## Results

### Resistance spectrum

Overall, 2,651 adult mosquitoes reared from larval collections from both groups of villages (‘low risk’ and ‘high risk’) were identified morphologically as *An. gambiae s.l* Of these, 2204 mosquitoes were exposed (220–222 individuals per insecticide) to the discriminating doses of DDT (4%), permethrin (0.75%), deltamethrin (0.05%), malathion (5%) and propoxur (0.1%). Field populations of *An. gambiae s.l* from both groups of villages showed resistance to DDT, permethrin, deltamethrin, and malathion. In contrast, these mosquitoes were highly susceptible to propoxur with a mortality rate of 99.5% in ‘low risk’ and 100% in ‘high risk’ villages within 60 minutes of exposure (Table 1). Difference in mosquito mortality rates between the two groups of villages for each insecticide treatment was not significant (p>0.05). Whereas, mortality in the laboratory strain of *An. arabiensis* was 100% to all the five tested insecticides. With DDT and permethrin no knockdown was observed within 60 minutes of the exposure period. The KDT_50_ values for deltamethrin were also high and similar in mosquito samples from both groups of villages (47.7 min for ‘low risk’ and 43.5 min for ‘high risk’). The KDT_50_ and KDT_95_ for the laboratory strain of *An. arabiensis* for DDT, permethrin and deltamethrin was 23.7 and 38.2, 17.2 and 24.1, and 16.8 and 25.2 minutes, respectively (data not shown).

**Table 1:** Mortality rate and knockdown in field populations of *Anopheles arabiensis* exposed to discriminating concentrations of 5 insecticides in Gilgel-Gibe dam area, southwestern Ethiopia.

Village group	Insecticide	n	KDT_50_ [95% CI]	KDT_95_ [95%CI]	% Knockdown at 60 minutes [95% CI]	Percentage mortality [95% CI]
‘Low risk’	DDT (4%)	220	__a	__a	0.9 [0.0, 2.5]	2.3 [0.0, 6.3]
	Permethrin (0.75%)	220	__a	__a	7.3 [1.8, 12.7]	26.4 [18.0, 34.7]
	Deltamethrin (0.05%)	222	47.7 [46.4, 49.1]	__a	78.8 [72.6, 85.0]	67.1 [61.4, 72.8]
	Malathion (5%)	220	*	*	*	81.8 [76.5, 87.1]
	Propoxur (0.01%)	220	*	*	*	99.5 [98.3, 100.0]
‘High risk’	DDT (4%)	220	__a	__a	0.0 [0.0, 0.0]	0.5[0.0, 1.7]
	Perimethrin (0.75%)	220	__a	__a	4.5 [2.5, 6.5]	20.8 [14.9.26.7]
	Deltamethrin (0.05%)	222	43.5 [41.0, 46.1]	__a	82.2 [74.1, 91.0]	55.1 [48.3, 61.8]
	Malathion (5%)	220	*	*	*	76.7 [66.9, 84.5]
	Propoxur (0.01%)	220	*	*	*	100.0 [100.0, 100.0]

n =  number of mosquitoes tested; __a = 50% knockdown was not obtained within the 60 min of exposure period as result, KDT_50_ and/or KDT_95_ could not be estimated;

*  =  Lack knockdown effect.

### Mosquito identification, *kdr* and ace-1^R^ mutations

All of the 463 *An. gambiae s.l* specimens randomly drawn from dead and alive individuals exposed to insecticide impregnated papers were molecularly identified by PCR as *An. arabiensis*. Of this, 284 and 176 mosquito specimens were molecularly screened for *kdr* and *ace-*1^R^, respectively. The L1014F-*kdr* allele was present in 280 (137 alive and 143 dead) specimens, and the remaining 4 specimens did not give results with the assay. The L1014S-*kdr* was not detected in the tested mosquito specimens from both groups of villages. The majority (96.4%) of the tested individuals was homozygous (RR) and 3.6% were heterozygous (RS) for L1014F-*kdr* allele. Allelic frequencies of L1014F-*kdr* in both alive and dead mosquito specimens from both groups of villages were in the range of 95–100% (Table 2). There is no evidence for the presence of association between the *kdr* genotype and resistance phenotype for permethrin and deltamethrin in mosquito specimens from both ‘low risk’ and ‘high risk’ villages (p>0.05), but test of association between *kdr* genotype and resistance phenotype could not be estimated for DDT as very few susceptible specimens were collected. The observed genotype frequencies in this population of mosquitoes did not deviate from the expected genotype frequencies predicted by the Hardy-Weinberg equilibrium (χ^2^ = 0.093, p = 0.76). Out of the 176 screened, 169 of specimens were scored for ace-1^R^ allele and no ace-1^R^ mutation (G119S) was detected. Likewise, of the 44 mosquito specimens randomly drawn from the laboratory strain of *An. arabiensis* and scored for *kdr* mutation, 41 carried the homozygous (SS) wild-type allele while 3 specimens did not give results with the assay. ACE-1^R^ mutation was not detected in all 32 randomly drawn dead laboratory strain of *An. arabiensis* specimens which were scored for the allele.

**Table 2:** *kdr* genotype frequencies in field populations of *Anopheles arabiensis* from southwestern Ethiopia according to their survival in the bioassay test.

Village group	Bioassay phenotype	Number assayed	Genotype			Allele frequency	
			RR	RS	SS	R	S
‘High risk’	DDT survivors	28	27	1	0	0.98	0.02
	DDT dead	ND	n/a	n/a	n/a	n/a	n/a
	Permethrin survivors	29	29	0	0	1.00	0.00
	Permethrin dead	29	27	2	0	0.97	0.03
	Deltamethrin survivors	28	28	0	0	1.00	0.00
	Deltamethrin dead	28	27	1	0	0.98	0.02
‘Low risk’	DDT survivors	29	28	1	0	0.98	0.02
	DDT dead	1	1	0	0	1.00*	0.00
	Permethrin survivors	27	27	0	0	1.00	0.00
	Permethrin dead	29	26	3	0	0.95	0.05
	Deltamethrin survivors	24	24	0	0	1.00	0.00
	Deltamethrin dead	27	25	2	0	0.96	0.04

RR  =  homozygous resistant; RS  =  heterozygous; SS  =  homozygous wild-type; ND  =  not done because the dead specimens were not fallen in the sampled specimens; n/a  =  not applicable;

*  =  Allele frequency over estimated because the only non-survivor specimen to DDT bioassay found to carry L1014F allele after genotyping.

### DDT residue inside human dwellings and in the environment

The mean concentration of DDT residue found in the indoor soil samples was higher in ‘high risk’ than the ‘low risk’ villages while no residue was found in the control village (Table 3). Moreover, DDT was not detected in all the environmental soil samples of study villages, and a control village. History of DDT spray of houses in ‘low risk’ and ‘high risk’ villages before the sampling date was in the range of 1 to 144 months. The mean concentration of DDT on the wall and floor surfaces of houses sprayed only in June 2008 (13 months before the sampling date) was 1.21±0.74 g/m^2^, and 0.14±0.24 g/m^2^, respectively. For houses sprayed in June 2008 and June 2009 consecutively, the concentration was 1.63±1.08 g/m^2^, and 0.47±0.30 mg/m^2^, respectively. The difference between the means of concentration of DDT on the walls and floors of houses was significant (P<0.05). Houses sprayed more than 13 months before the sampling date were excluded from the analysis because they were few in number per spray programme for comparison.

**Table 3:** Concentration of DDT residue in mg/kg of soil and mg/m^2^ surface area in soil samples from wall and floor surfaces of houses and the environment among seven villages in Gilgel-Gibe area, southwestern Ethiopia.

Village group	Site of sample collection	N	Mean ± SD (mg/kg/soil)	Mean ± SD (mg/m^2^ surface)
‘Low risk’	Wall surface	22	36.87±90.75	0.21±0.56
‘High risk’	Wall surface	45	316.54±236.71	1.45±1.16
‘Low risk’	Floor surface	22	2.59±4.88	0.02±0.03
‘High risk’	Floor surface	38	60.86±72.17	0.30±0.32
Control	Wall surface	11	n/d	n/d
Control	Floor surface	11	n/d	n/d
‘Low risk’	Environment	21	n/d	n/d
‘High risk’	Environment	44	n/d	n/d
Control	Environment	11	n/d	n/d

n  =  number of soil samples; SD  =  standard deviation; n/d  =  not detected.

## Discussion

Results of this study showed that field populations of *An. arabiensis* collected from the two groups (‘low risk’ and ‘high risk’) of villages developed resistance to DDT, permethrin, deltamethrin and malathion but not to propoxur. Similar observation of resistance in populations of *An. arabiensis* to DDT, permethrin and malathion had been reported from Sudan [20]. The high susceptibility of the mosquito population to propoxur is also in agreement with a study conducted in Sudan where populations of *An. arabiensis* were susceptible to bendiocarb, a carbamate insecticide [20].

The development of resistance by the mosquito population to DDT as well as to type I and type II pyrethroids could jeopardize the current malaria control programs. The reduced susceptibility of the mosquito population to malathion would affect the use of organophosphates as alternative insecticides to DDT in IRS as well. Thus, currently, carbamate insecticides are the only alternative to be used, at least in this region of the country, in IRS. However, their short residual effect and their toxicity make them inappropriate for impregnation of bednets [42]–[45].

According to our data (Table 2), no significant association was observed between the *kdr* mutation (L1014F) and resistance phenotype (P>0.05) which is in agreement with previous studies [16], [39], [46]–[48]. This could be due to a variation in *kdr* allelic expression. L1014F-*kdr* mutations determining genotype-resistance phenotype relationship in *Culex quinquefasciatus* goes through transcriptional regulations responsible for the discrepancy of monoallelic or biallelic variation of gene expression in *kdr*-mediated resistance [49]. Consequently, homozygous resistant individuals may express the susceptible allele and the homozygous susceptible individuals could express the resistant allele [50]. The occurrence of the resistant allele in both the dead and alive specimens suggests that *kdr* mutation may exist with another factor necessary for the expression of the resistance phenotype, and resistance could also be multigenic as a result *kdr* may not fully explain all the variance in phenotype [48], [51]–[52]. Bioassay non-survivors to DDT and pyrethroids were found with homozygous resistant alleles (L1014F/L1014F) which suggest that besides the L1014F *kdr* mutation other mutations in the para-type sodium channel gene might be needed for mosquitoes to survive an exposure to a discriminating concentration of an insecticide [53]. Knockdown resistance mutation may also provide complete resistance to doses of field spray but not to WHO discriminating concentrations. Hence, further investigations are required to determine the role of *kdr* in conferring resistance and the presence of other resistance mechanisms involved in the different classes of insecticides. This may include employing other methods of genotyping such as sequencing, and new assays which may help to evaluate possible method related confounding factors in allele scoring to determine genotype–phenotype association [54]–[55].

The degree of resistance conferred by L1014F-*kdr* varied between DDT and pyrethroids. The presence of a single *kdr* allele provided a resistance advantage against DDT compared to pyrethroids which is in agreement with findings of a similar study [56]. Though it is difficult to make valid comparison owing to few numbers of mosquitoes with RS genotype from DDT and pyrethroid bioassays, the homozygous resistant genotype (RR) seemed to confer higher resistance than the heterozygous (RS) against pyrethroids but not to DDT. Hence, it may be difficult to predict the level of dominance of a resistance gene unless the precise physiological role of this gene and its mode of interaction with the insecticide are known [44]. The absence of ace-1^R^ mutation (G119S) in both dead and alive specimens exposed to malathion or propoxur, and the absence of cross-resistance between these two insecticides further suggests the existence of other metabolic resistance mechanisms that could confer resistance only to malathion [57].

The low mortalities (0.5–2.3%) of *An. arabiensis* after exposure to the discriminating concentration of DDT suggest a high level of insecticide resistance resulting probably of prolonged and extensive use of DDT for IRS by the NMCP in Ethiopia. Indeed, DDT residues were only found inside the houses and not in the surrounding environment. Other previous studies also showed that the selection of resistance to DDT in the populations of malaria vectors was due to the long-standing and extensive use of DDT in the IRS program [58]–[60]. The reduced susceptibility of these mosquitoes to malathion could also be attributed to use of malathion as an alternative insecticide in the IRS programme of Ethiopia. In agreement with this study, *An. arabiensis* (already resistant to DDT) became resistant to malathion after one year of house treatment as an alternative to DDT in the irrigation schemes of Gezira in Sudan [61].

The endophilic resting behaviour of population of *An. arabiensis* of the study area ([62], D Yewhalaw unpublished data) further suggests that their exposure to DDT, pyrethroids and malathion could be indoor instead of outdoor. Hence, resistance selection in the mosquito population to DDT and pyrethroids (permethrin and deltamethrin), and the reduced susceptibility to malathion seemed most likely to have been developed as a consequence of indoor exposure of adult mosquitoes to these insecticides from IRS and ITNs/LLINs. Resistance selection in *An. arabiensis* from Gezira, Sudan, was also presumed to occur at the adult stage as a result of IRS to control malaria vector [21].

Moreover, despite the regular spray, the mean concentration of DDT found on the walls of houses was less than 2 g a.i./m^2^ of surface, the target application rate set by WHO [63]. This shows that under-dosage (poor management of insecticides) might have contributed to the development of DDT resistance [64]–[65].

In conclusion, the observed multiple-resistance coupled with the occurrence of high *kdr* mutation frequency in populations of *An. arabiensis* could profoundly affect the current malaria vector control programme in Ethiopia. This needs an urgent call for implementing rational resistance management strategies and integrated vector control intervention.
